# Molecular docking analysis of flavonoid compounds with HIV-1 Reverse transcriptase for the identification of potential effective inhibitors

**DOI:** 10.6026/97320630015646

**Published:** 2019-10-12

**Authors:** My Abdelaziz El Alaoui, Souad El Fartah, Najwa Alaoui, El Mostapha El Fahime, Amar Habsaoui

**Affiliations:** 1 Functional Genomic Platform, UATRS, Center for Scientific and Technical Research [CNRST], ZIP 10000 Rabat, Morocco; 2 Laboratory of Genetics and Biometry, Faculty of Sciences, Ibn Tofail University, Kenitra, Morocco; 3 Research Team in Molecular Virology and Oncobiology, Faculty of Medicine and Pharmacy, University Mohammed V in Rabat, Av. Mohamed Belarbi El Alaoui, ZIP 6203, Rabat, Morocco; 4 engineering and environment laboratory, Modeling and Application LIMEMA, Faculty of Science - University Ibn Tofail ,Kenitra .B.P 133 Kenitra 14000, Morocco

**Keywords:** Flavonoid, Drug design, Reverse Transcriptase

## Abstract

It is of interest to elucidate the binding mode analysis of 18 sulphonated flavones in the non nucleoside inhibitory binding pocket of the HIV-1 reverse transcriptase (PDB ID: 1RTD). We further compared them with the known Non Nucleosidic Reverse Transcriptase Inhibitors (NNRTI) drug molecules such as delaviridine, nevirapine and etravirine. Molecular docking studies of sulphonated flavones were performed in the binding pocket of reverse transcriptase using the PatchDock server. The flavones have different binding energies with RT and the atomic contact energy (ACE) value of sulfonated flavones range from-389 to-231 Kcal/mol while docking of the commercialized NNRTI showed the ACE value range from -486 to -224 Kcal/mol. This shows that most sulfonated flavones have ACE similar to the known NNRTI. Thus, seven compounds (FS-6, FS-7, FS-8, FS-9, FS-14, FS-15, FS-17) were reported as potent, selective, orally bio available, and nontoxic lead based on ADMET screening and effective binding analysis in the active site of the reverse transcriptase (PDB ID: 1RTD) for further consideration. We further document that compounds (FS-1, FS-10, FS-4 and FS-12) have unfavorable binding features to be considered as leads.

## Background

**Figure 1:**
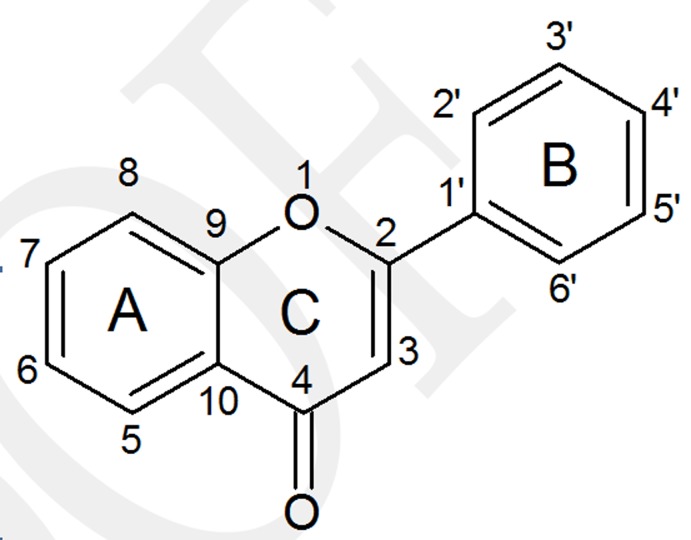
The skeleton structure and numbering of the flavones, with rings named and positions numbered

Flavones are a subclass of natural compounds belonging to the class of flavonoids which are secondary metabolites very common in plant kingdom and responsible for numerous biological activities. All the flavonoids are derived from the benzo-γ-pyrone and can be classified according to the nature of the different substituents present on the cycles of the molecule and the degree of saturation of the bezo-γ-pyrone skeleton, their basic backbone is formed of 15 carbon atoms grouped into a phenyl ring (B) and a benzopyrone ring (A and C) [1] as shown in Figure 1. In natural environment, the majority of flavones exist as the O-glycosylated or rarely C-glycosylated form, but we can also find the aglycone or genin that are often hydroxylated and/or methoxylated. Flavonoids are polyphenols compounds which are classified as flavonols, Flavones, flavanols, isoflavones, flavanones, catechins and anthocyanidins [2]. The reactivity of flavones and their physiological effects are linked to their structures and to the nature and the complexity of the constituents fixed on the basic backbone [3,4]. So far many studies were carried out for the synthesis of various functionalized flavonoids; these studies were also focused to improving their aqueous solubility and thus their bioavailability. In addition, previous research showed that the mechanism of action of flavones are related to their ability to scavenge free radicals but also to complexing essential metals for the catalytic activity of enzymes [5,6]. Furthermore, the pharmaceutical efficiency of the sulfonated compounds depends on their ability to bind specifically to target protein. To better understand the mechanisms of interactions between sulphated macromolecules and amino acids Ojada et al have studied these interactions using X-ray crystallography [7]. Flavonoids are becoming the focus of medical research, they were used in many therapeutic application, such as anti-inflammatory, antimicrobial and anti-carcinogenic agents, others flavonoids were confirmed to be as inhibitors of numerous enzymes such as DNA synthetases and RNA polymerases, they have been also reported to present an excellent inhibition of human immunodeficiency virus (HIV-1) Protease (PR) and Reverse Transcriptase (RT) [8]. Previous studies have shown that some methoxylated flavones in position 3 have significant antiviral activities [9], citing for example the 4',7-dihydroxy -3,5,6-timethoxyflavone tested in vitro have a significant antiviral activity against the poliovirus type 1 and rhinavirus type 15 [10], on the other hand, studies have shown that flavonoids carrying numerous hydroxyl groups, especially in position 5' and 4' (Apigenin, Myricetine...), exhibit very significant inhibitory activities for HIV1-RT with IC50 values less than 100 µg/mL [11], the baicalein (5,6,7-trihydroxyflavone) tested also on the HIV-1 RT exhibited a 90% inhibition of the enzyme at a concentration of 2µg/ml [12]. Katsuhiko et al found that polyhydroxylated flavones have significant inhibitory activities against HIV1-RT, it was inhibited by 100%, 90% and 70% in the presence of 2 µg/mL of quercetin, myricetin, quercetagetin and baicalein, respectively [13]. The xanthohumol, a flavonoid extracted from Humulus lupulus, was tested also for its HIV-1 RT inhibitory activity by the Yong-Tang Zheng et al, the results showed a significant inhibitory potency at concentrations of approximately 24 µM [14].

The reverse transcriptase is a very interesting target for anti-HIV treatments, because it plays a central role in the viral replication and acts at the primary stage of infection whereas only two copies of gRNA are available. HIV-1 RT is known for its reverse transcription activity, nuclease activity (RNAse H) and DNA polymerase activity [15] HIV-1 RT is a multifunctional dimer formed of two subunits P51 and P66 with a molecular mass of 51-kDa and 66-kDa respectively. The P66 larger subunit is composed of 560 amino acids and includes both catalytic sites of the enzyme (RNAse H and DNA polymerase activity), on the other hand the P51 subunit it has no enzymatic activity but has an important structural role.

There are many antiviral therapeutic agents used for the inhibition of HIV-1 RT those which act at the catalytic site are called Nucleosidic Reverse Transcriptase Inhibitors (NRTI) on the other hand those which act via the allosteric site are referred as Non Nucleosidic Reverse Transcriptase Inhibitors (NNRTI). Treatment with Non-nucleoside compounds and nucleoside analogues are being used as reverse transcriptase inhibitors, but due to the emergence of drug resistance mutations [16-20], researchers continue to develop and test new promising natural products such as flavones used as reverse transcriptase inhibitors [9]. Several resistance mutations have been described against commercialized drugs, K103N is the mutation associated to a resistance to NNRTI which is most often encountered in vivo [21], the mutation Y181C conferred a strong resistance to Nevirapine and Delavirdine, while the Y188L increases the IC50 of a factor of thousand to Efavirenz [20], Whereas the M230L mutation resulted in low resistance to the three commercialized drugs (Nevirapine, Efavirenz Delavirdine), Finally the mutation P236L is relatively rare but causes a strong resistance to Delavirdine.

The objective of this study was to elucidate the binding mode analysis of 18 novel sulphonated flavones in the non nucleoside inhibitory binding pocket of reverse transcriptase (PDB ID: 1RTD) and to compare them with the commercialized NNRTIs drug such as Delaviridine, Nevirapine and Etravirine. The Docking study will gives a new insight into the investigation of molecular interaction between sulfonated compounds and allow us subsequently to select one or more best active flavones that will be the subject of an in vitro test in a future study.

## Methodology

### Preparation of the protein structure

The three dimensional structure of HIV-1 reverse transcriptase (PDBID: 1RTD) was retrieved from protein data bank (http://www.pdb.org) at 3.2Å RMSD resolution. The protein molecule were prepared mainly using VEGA ZZ software [22], the preparation process involves several steps: Adding hydrogen bonds, deleting water molecules and co-crystallized DNA-primer complex, than the active site residues within a range of 3.5 Å were selected and saved in PDB format for energy minimization. Before energy minimization, the atom constraints was applied the protein backbone and the Magnesium Ion were fixed to avoid any modifications in the experimental structure. After, the active site residues were minimized using NAMD with a 50000 steps conjugate gradients minimization. After refinement of the structure for correct formal charge the active site residues were saved as a PDB file, which will be used for the docking study.

### Preparation and optimization of ligands structures

A total of 18 sulphonated flavones (Table 4) were drawn using ACD/ChemSketch version 12 [23]. The 2D structures of sulphonated flavones were generated and the SMILE notations of the compounds were generated as well as the MOL2 format files, to convert the generated MOL2 files to PDB format we used the OPEN BABEL software [24] and for 3D structure refinement we used GlycoBioChem PRODRG2 Server [25], this operation allows us to select the lowest energy conformer for each compound.

### Protein-ligand docking

In silico docking studies were performed using PatchDock server [26,27], which uses the local geometric characteristics of proteins by limiting the search to certain areas of the molecular surface. For this, in a first step, the molecular surface undergoes a segmentation process using a segmentation algorithm for detection of geometric patches (concave, convex and flat surface pieces). These patches are then connected to form a three-dimensional graph, which includes convex, concave and flat patches then the surface patches of the protein are matched to surface patches of the ligand molecules. The complexes obtained are then reclassified by a score function depending on the geometric fit and atomic desolvation energy.

### Drug likeness and ADMET prediction

Drug-likeness was calculated with OSIRIS Property Explorer [28] (http://www.organic-chemistry.org), which predicts for pharmaco kinetic descriptors including drug likeness, drug score, mutagenicity, irritancy, and reproductive effect. The in silico ADMET profiling of a drug molecule which constitutes the pharmacokinetic profile and its possible effects on health such as blood/brain barrier penetration, HIA (Human Intestinal Absorption), Caco-2 cell permeability and aqueous solubility. In this study, we have used the ACD/Labs I-lab 2.0 web-based application (https://ilab.acdlabs.com/iLab2/).

### Molecular Modelling and graphical visualization

The frontier orbitals HOMO (Highest Occupied Molecular Orbital) and LUMO (Lowest Unoccupied Molecular Orbital) calculations were performed with ArgusLab 4.0.1 program [29] graphical displays were generated with PyMol Molecular Graphic System Version 1.7.4.5 Edu and LigPlot+ [30].

## Results and Discussion:

### Structure of the sulfonated flavones

Before running molecular docking simulation, the 3D structure of flavones compound obtained from glyco- BioChem PRODRG2 Server [25], were assessed for their geometry and molecular conformation in comparison with published structures, the conformational analysis of flavonoids show that the dihedral angles between both plans of flavones (plane of the ring A, C and B) is close to 0°. Despite the steric hindrance, the delocalization of the π electron cloud tends to maintain the molecule in the plan. This co planarity is observed in most studies, but some authors have found angles of about 40° [31,32] this demonstrates that the obtained conformers were of very good quality.

### Molecular Modeling

In order to gain insight into the interaction modes of the studied flavones, the frontier orbitals HOMO and LUMO were used to measures the electron donor character and acceptor, this energy is represented in the (Figure 3), The molecular orbital shows that the contribution of HOMO is especially located on the core B comprising the sulfonyle group (Sulfonate) in position 5' which is responsible for electron transfer between molecules and target key residue in the HIV1-RT. The predominant acceptor character of the flavones presented in our study relative to the donor character explains their ability to interact with the amine function of target key residues of the HIV1-RT. In the same context, the study conducted by Gaydou et al [33-35] has allowed synthesizing tens of sulfonated flavones some of which, their structures were resolved by X-ray diffraction, It was also demonstrated that these sulfonated flavones have the ability to form a complexes with amino acids such as the paratoluidine, as well as a complexation of nucleic acids [34,36]. The flavonoids which form a complexes with amino compounds in an aqueous medium reflect of their potential to interact with the proteins, this is due to the sulfonic acid group of flavonoids who is bioisostere of the carboxylic function which plays a predominant role in the interaction between this group and any target amino acid of the protein, thus, the reactivity pattern that occur with flavones compounds is due to the labile proton of the acid function which binds to the amine function of amino acids. This finding corroborates with the LUMO and /or HOMO states attributed to the sulfonic acid group in the studied compounds.

### Molecular docking studies

In silico, docking was carried to improve our knowledge of the interactions patterns between the reverse transcriptase (PDBID: 1RTD) and the studied sulfonated flavones, the docking results are summarized in the Table 1. The atomic contact energy (ACE) value of sulfonated flavones range from -389 to -231 Kcal/mol while docking of the commercialized nonnucleoside reverse transcriptase inhibitor (NNRTI) showed the ACE value range from -486 to -224 Kcal/mol, this result reflects that most sulfonated flavones have ACE similar to the most commer- cialized NNRTI. The highest atomic contact energy predicted for NNRTI was observed in the Delaviridine and CP 94707 [21] with the ACE values of -486 and -403 Kcal/mol respectively, while all other NNRTI showed energy lower than the ACE value of the studied sulfonated flavones, the Rilpivirine, Nevirapine, Mulberrin, Etravirine, Vitexilactone and efavirenz showed ACE value of -363, -340,-337,-282,-228 and -224 Kcal/mol respectively. The sulfonated flavones having highest atomic contact energy were ranked after delaviridine and CP 94707 with ACE value of -389,-377, -370 and -370 Kcal/mol and which are attributed to FS-15, FS-17, FS-11 and FS-18 respectively.The rest of the flavones were ranked according to their ACE values in the eighth and twentieth position compared to Etravirine, Vitexilactone and efavirenz.Thus from the predicted ACE values it can be inferred that the studied sulfonated flavones presented a favorable binding energy with the HIV-1 reverse transcriptase (PDBID: 1RTD) in particular the FS-18, FS-8 and FS-10

To gain insight on the binding modes of studied flavones as per the molecular docking results, visual poses inspection analysis has been performed for sulfonated flavones which exhibited highest ACE value against the active site pocket of the HIV-RT (PDBID: 1RTD) taking into account the presence of hydrogen bonding and the length of their interaction with the key residues of the HIV-RT in the active site pocket. Among the residues which play a crucial role in the mechanisms of action of NNRTI we find the Tyr181, Tyr 183, Tyr 188, Tryp W229 and Lys103, other residues that are common sites of NNRTI in the binding pocket we can cite, in particular the Leu-100, Lys-101, Pro-225, Pro-236, Val-106 and Val-101 [37]. The mechanism of action of NNRTI inhibitors is mediated by changing the shape of the pocket, other NNRTI inhibitors also act in part by indirect modification in the pocket structure [38]. In the study conducted by Janice D. Pata et al [21] , the structure of the HIV-1-RT complexed with CP-94,707 which is an active compound against mutations in Tyr-181, Try-188, and Lys-103 in the hydrophobic pocket of the HIV-1-RT. In this study the authors demonstrate that most of the mutations that confer drug resistance directly interfere with inhibitor binding by changing the shape of the binding pocket.

**Figure 2:**
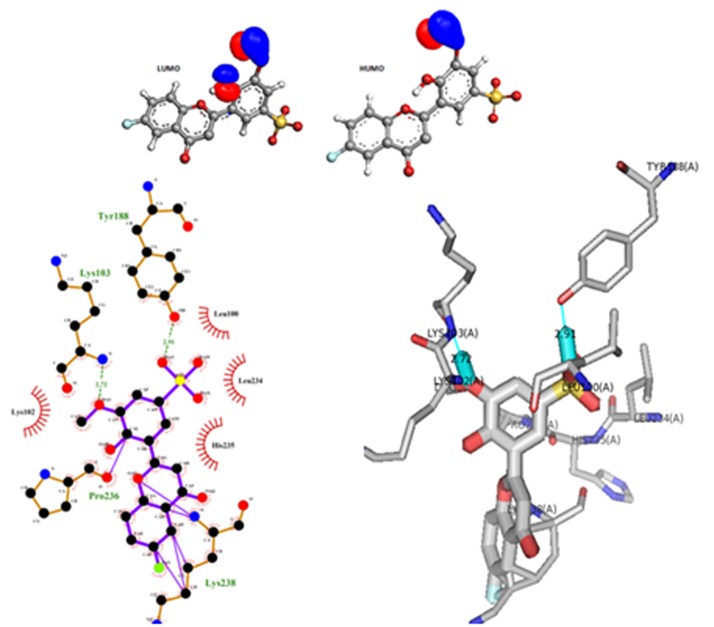
Binding modes of FS-6compound with HIV-1 RT (PDBID: 1RTD) visualized using LigPlot+ and pymol software, this Flavone exhibit an ACE value of - 224 Kcal/mol and showed two hydrogen bond interactions (green dash lines) with Tyr 188 (2.91 Å) and Lys 103 (2.72 Å) as well as five covalent bonds (purple lines) with the Lys238 and one covalent bonds with the Pro236.

**Figure 3:**
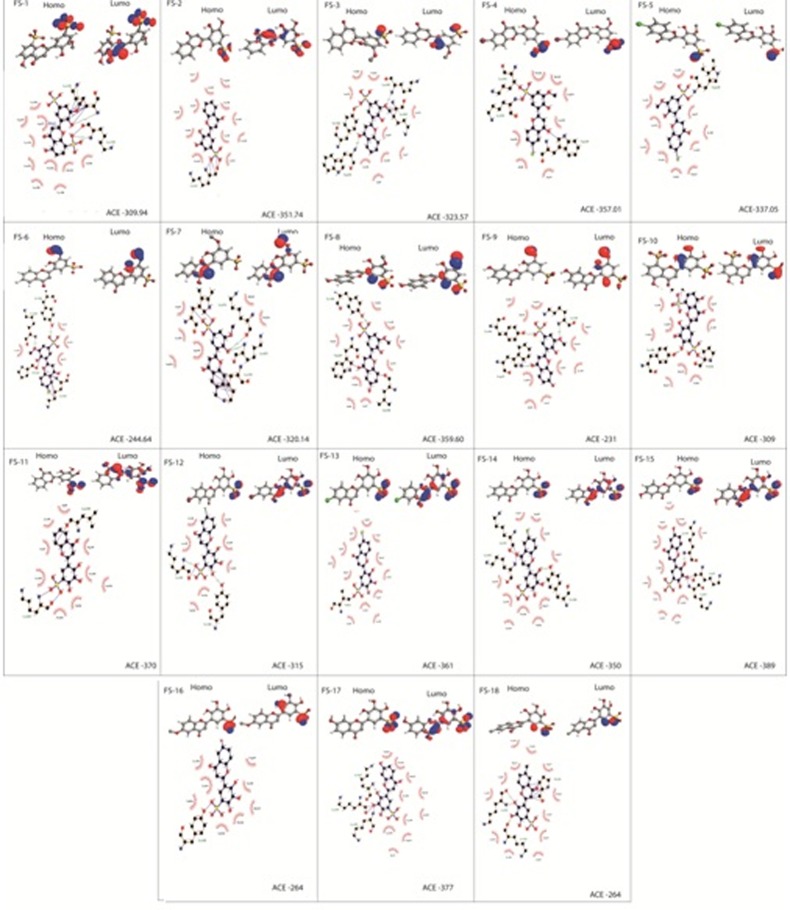
Binding modes and the frontier molecular orbitals for the studied sulfonated flavones is shown. The molecular orbital shows the location of possible sites responsible for electron transfer between molecules and the key residues of the reverse transcriptase (PDBID: 1RTD), the ACE values for each flavone are also given

The interactions of each sulfonated flavones with the key residues of the HIV-1 reverse Transcriptase demonstrated that all the flavone interact with most the residues of the hydrophobic pocket as shown in (Figure 3). Our results demonstrate that all studied compounds have hydrogen bonds with at least one key residue of the enzyme, the most represented are Tyr 188, Lys 103, Lys 101, Trp 239 and Pro 236 and the distances of hydrogen bonds vary between 2 Å and 3 Å, other covalent bonds come to reinforce the interactions were observed in the following compounds: FS-2, FS-3, FS-4, FS-6, FS-7 (Figure 3). Compound FS-9 had 3 hydrogen bond interactions with the key residue of the HIV-1-RT, the Tyr188 at a distance of 2.17 Å, Lys103 at a distance of 2.95Å and the Trp239 at a distance of 2.26Å. Also, compound FS-1 got 3 hydrogen bond interaction with the Lys 103 at a distance of 2.18 Å, Lys102 at a distance of 2.4 Å in addition to hydrogen bond this compound is stabilized by tow covalent bonds between Lys 101 , Lys 102 (Figure 3). The FS-6, illustrated in Figure 2, has two hydrogen bonds with Tyr 188 (2.91 Å) and Lys 103 (2.72 Å) as well as five covalent bonds with the Lys 238 and one covalent bonds with the Pro 236, in this interaction, the phenolic hydroxyl of the tyrosine 188 form a hydrogen bonds between one of the oxygens (S=O) of the sulfonyl group of the flavone. The other hydrogen bonds are created between the hydrogen of the alpha amino group of Lysine 103 and the oxygen of the methoxy in 3' position of the B core of the flavone (Figure 1).That case seems obvious, because for the flavones, the methoxy in position 3' are oriented out of the plane of the cycle, and the Electron density is important at this oxygen, this promotes the establishment of hydrogen bonds. The frontier orbitals HOMO and LUMO measured for this flavone (Figure 2) has allowed us to have an additional argument to explain the location of the resulting interactions. The HOMO confirms the high electron density in the oxygen of the methoxy in 3' which is favorable to form a hydrogen bond with the Lys103 with an ACE value of - 224 Kcal/mol.

In their article, Hopkins et al. [39] established a distance map of ligand-receptor interaction from a crystal structures of HIV-1 RT complexed with two potent inhibitors, MKC-442 and TNK-651 at 2.55 Å resolution, it was demonstrated that amino acids considered in the interaction are at 3.3 Å from MKC-442, these amino acids are: L100, K101, V106, V179, Y181, Y188, G190, F227, W229, L234, H235, P236 et Y318. In addition most important mutations of HIV- 1 RT occur in amino acids Lys-103, Tyr-181, and Tyr-188 [21]. In our study FS-9, FS-17, FS-3 (Figure 3) was found to be forming a hydrogen bond interactions with the Tyr 188 and the Lys 103 with an ACE values of -231, -377, -323 Kcal/mol respectively, this compound can be considered as new drugs that are selectively act on HIV1-RT taking into account drug-resistance mutations in the cited residues. In addition the interaction of the other sulfonated flavones with the key residues of the p66 subunit of HIV-1 RT (PDBID: 1RTD) showed different ACE binding energies and occupy the active site unlike that of the commercialized NNRTI (Figure 3).

### In silico ADMET prediction

Among the criteria that define a good drug, we find its absorption, distribution, metabolism, excretion and toxicity (ADMET), this descriptors and pharmaceutically relevant properties helps to evaluate biologically activity molecules and eliminate biologically inactivate one according to Lipinski rule of Five [40]. In our study we have analyzed pharmacokinetics descriptors (ADME) of sulfonated flavones using admet SAR toolbox [41] and the toxicity assessment for lethal dosages and probability of health effects using ACD/ I-Lab 2.0 (guest) and OSIRIS Property Explorer [28]. The results from OSIRIS Property Explorer shown that all the studied flavones were found to be in accordance of Lipinski’s Rule of Five (Ro5) except for tow flavones, who does not respect the Lipinski Ro5 because the number of hydrogen bond acceptors (HBA) is greater than 10, the concerned flavones are: FS-1 and FS-10 (Table 5), the HBA was 11 and 12 respectively, in addition two others flavones which present mutagenic effect were observed FS-4 and FS-12. However the studied compounds represent good solubility, bioavailability and have no reproductive, no irritant and no tumorigenic effect. Further pharmacokinetic properties were used to select the best drug candidate (Table 2) among which were PlogBB (blood/brain), logHIA (intestinal barrier), Pcacoc (cell permeability), logpGI (substrate/non-Inhibitor), PlogS (aqueous solubility) and Logpapp (cell permeability), all sulfonated flavones showed significant values for the properties analyzed within acceptable range in comparison with the commercialized NNRTI (Table 2) except for some flavones FS-11, FS-12, FS-13, FS-14, FS-18, FS-15, FS-16, FS-17 which exhibit low blood barrier penetration (PlogBB) for central nervous system (CNS) activity and low absorption across intestinal barrier (logHIA) (Table 2), while for predicted cell permeability (PCaco-2), all tested flavones shows to be in the acceptable range ,which reflects a good permeability across intestinal barrier and help in good transport of drug metabolic compounds.

The logPGI Drug-drug interactions (DDIs) within tissue that transforms xenobiotics of vigorous reduction drug absorption and released more bile (liver) and urine (kidney) [42]. The acceptable range of-5 (poor) to +1 (good) and substrate inhibitor from 0 to 1 in which the commercialized NNRTI and tested flavones shows good activity with human intestinal absorption and metabolism. For the aqueous solubility (PlogS) of sulfonated flavones came within acceptable arrange which reflect their good solubility. The toxicity of sulfonated flavones was assessed based on lethal dosage (LD50) and functional probability effect on different tissues in rat model. The LD50 mouse and probability of health effects were predicted using ACD/I-Lab 2.0 (guest). The toxicity of selected flavones was summarized in the (Table 3).The lower the dose the more toxic the compound, from our results (Table 3) we found that tested flavones have less reliability on oral, subcutaneous, intra peritoneal, and intravenous when compared to reference molecule, the reliability index lie between 0.47-0.12 borderline which reflect that some tested flavones (e.g., FS-1, FS-3, FS-11, FS-14, FS-16) were within the border line effect on reliability index and others were observed note reliable (Table 3) The toxicity and health effects predicted for blood, cardiovascular system, gastrointestinal system, kidney, liver, and lungs within the therapeutic dose range revealed that the sulfonated flavones FS-6, FS-8, FS-9, FS-18, FS-15, FS-17, FS-14 are less toxic on blood, cardiovascular system, gastrointestinal system, kidney, liver, and lungs, respectively and no side effect was observed in the tested dosages.

## Conclusion

Flavones are a subclass of natural compounds belonging to the class of flavonoids. They are widely studied for their ability to inhibit several enzymes such HIV-1 reverse transcriptase (RT), protease (PR), and integrase (IN). It is of interest to select such compounds as lead molecules with optimal binding features for RT (PDBID: 1RTD at the sites of Tyr 188, Lys102, Lys103 and Tryp 239) using molecular docking and ADMET/SAR screening. Thus, we report seven compounds (FS-6, FS-7, FS-8, FS-9, FS-14, FS-15, FS-17) as potent, selective, orally bioavailable, and non toxic leads based on the ADMET screening and effective binding analysis in the active site of the reverse transcriptase (PDBID: 1RTD) for further consideration.

## Disclosure:

This research did not receive any specific grant from funding agencies in the public, commercial, or non-profit sector.

## Figures and Tables

**Table 1:** Interactions of the studied flavones with the key residues of the RT enzyme (PDBID: 1RTD) Hydrogen and Ligand Bound are shown, the energies of the atomic contact (ACE) and Patch Score are represented equally

Compound ID	H-Bound	Amino Acids	Ligand Bound	PatchScore	ACE
FS-9	3	Tyr 188 (2.17 Å), Lys103 (2.95Å), Trp 239 (2.26Å)	-	4438	-231
FS-6	2	Tyr 188 (2.91 Å), Lys 103 (2.72 Å)	Lys238 , Pro236	4270	-244
FS-1	3	Lys 103 (2.18 Å), Lys102 (2.4 Å)	-	4628	-309
FS-12	2	Tyr 188 (3.10 Å), Lys103 (2.37Å)	-	3964	-315
FS-3	3	Trp 239 (2.82 Å), Tyr188 (2.01 Å), Lys103 (2.07Å)	Lys102	4168	-323
FS-7	2	Lys 103 (3.10 Å), Lys101 (3.07Å)	Trp239, Lys102	4266	-320
FS-14	1	Tyr 188 (2.76 Å)	Lys101,Lys102	4056	-350
FS-5	1	Trp 239 (2.08 Å)	-	4490	-337
FS-10	1	Tyr 188 (2.25 Å)	Pro 236, Trp 239	4230	-350
FS-8	1	Tyr 188 2.29 Å	Trp 239, Leu100	4380	-359
FS-18	3	Lys 101 (2.11Å), Lys103 (2.47Å-2.21Å)	Pro 236	4456	-370
FS-17	1	Lys 103 (2.10 Å)	Lys 101, Lys 102	4136	-377
FS-15	1	Lys 103 (2.27Å)	Lys 101, Lys 102	3998	-389
FS-16	-	-	Tyr 188	4660	-264
FS-2	-	-	Lys 103	3886	-351
FS-4	-	-	Lys 102, Lys 103,Trp 239	4138	-357
FS-13	-	-	Val 106	4024	-361
FS-11	-	-	Leu 100, Lys 103	4166	-370

**Table 2:** ADME and pharmacological parameters prediction for sulfonated flavones using admet SAR toolbox

Compound ID	PlogBB^a^	logHIA^b^	Pcaco^c^	logpGI^d (substrate)^	logpGI^e (non-Inhibitor)^	PlogS^f^	Logpapp^g^
CP 94707	0.9846	0.9954	0.6333	0.6022	0.7596	-3.5107	1.4762
efavirenz	0.9182	0.9964	0.5553	0.7617	0.9557	-4.5228	1.758
Etravirine	0.7806	0.985	0.5389	0.5748	0.8911	-3.8854	1.1643
FS-1	0.6297	0.7737	0.6345	0.5539	0.7107	-3.5342	0.2321
FS-10	0.5875	0.5086	0.6987	0.6193	0.8288	-3.3488	-0.2215
FS-2	0.6661	0.6695	0.7025	0.6311	0.8128	-3.3794	-0.1651
FS-3	0.7703	0.8565	0.6246	0.6277	0.767	-3.5395	0.3814
FS-4	0.7218	0.8316	0.5915	0.6263	0.7555	-3.9848	0.4796
FS-5	0.702	0.8771	0.5852	0.636	0.6101	-4.0656	0.4779
FS-6	0.7584	0.8766	0.5891	0.617	0.6095	-3.89	0.4989
FS-7	0.7555	0.8206	0.6108	0.6294	0.7942	-3.5821	0.4868
FS-8	0.6297	0.7737	0.6345	0.5539	0.7107	-3.5342	0.2321
FS-9	0.6297	0.7737	0.6345	0.5539	0.7107	-3.5342	0.2321
FS-11	0.5875	0.5086	0.6987	0.6193	0.8288	-3.3488	-0.2215
FS-12	0.5449	0.5571	0.6475	0.6189	0.8133	-3.7858	-0.0633
FS-13	0.5181	0.5326	0.6453	0.6291	0.8872	-3.8953	-0.0698
FS-14	0.5988	0.5326	0.6459	0.6095	0.8912	-3.7036	-0.0456
FS-18	0.5083	0.6291	0.6746	0.5376	0.8511	-3.3664	-0.0978
FS-15	0.5875	0.5086	0.6987	0.6193	0.8288	-3.3488	-0.2215
FS-16	0.5708	0.5992	0.6369	0.5062	0.5931	-3.3908	-0.0812
FS-17	0.5875	0.5086	0.6987	0.6193	0.8288	-3.3488	-0.2215
Mulberrin	0.5317	0.988	0.7265	0.7565	0.8243	-3.6215	1.3303
Nevirapine	0.8002	0.7249	0.5418	0.6095	0.6714	-3.2026	0.3265
Rilpivirinezinc	0.844	0.9532	0.5795	0.6481	0.5642	-2.7706	1.23
Vitexilactone	0.8679	0.9776	0.651	0.8047	0.8792	-4.7862	0.7659
Delavirdine	0.6449	1	0.6231	0.7103	0.5644	-3.4929	0.7136

**Table 3:** LD50 ADME-TOX parameters and probability of health effects of sulfonated flavones using ACD/ I-Lab 2.0 (Algorithm Version: v5.0.0.184)

Compound ID	ADME-TOX parameters						System effect			
	Intra-peritonealnel^a^	Oral^a^	Intra-venous^a^	Subcu-taneous^a^	Blood effect^b^	Cardio-vascular^b^	Gastro-intestinal ^b^	Kidney^b^	Liver^b^	Lungs^b^
CP 94707	450 (0.43)	530 (0.44)	48 (0.36)	150(0.34)	0.37	0.62	0.85	0.38	0.19	0.75
FS-1	200 (0.19)	3000 (0.36)	580(0.35)	1100(0.43)	0.58	0.09	0.41	0.51	0.34	0.33
FS-10	500 (0.45)	1900(0.23)	500(0.43)	100(0.24)	0.83	0.73	0.09	0.53	0.15	0.39
FS-2	140(0.45)	1500(0.36)	160(0.38)	89(0.4)	0.53	0.35	0.07	0.28	0.28	0.16
FS-3	360(0.43)	1600(0.17)	280(0.25)	1000(0.38)	0.38	0.62	0.16	0.28	0.35	0.14
FS-4	390(0.43)	1300(0.21)	310(0.25)	1700(0.36)	0.32	0.05	0.65	0.37	0.33	0.13
FS-5	440(0.41)	1600(0.21)	300(0.28)	1400(0.37)	0.45	0.64	0.28	0.32	0.35	0.14
FS-6	450(0.41)	1400(0.19)	340(0.26)	2000(0.35)	0.52	0.61	0.33	0.3	0.39	0.55
FS-7	340(0.43)	1400(0.2)	240 (0.27)	990 (0.38)	0.43	0.62	0.28	0.29	0.41	0.13
FS-8	300(0.25)	1000(0.33)	300(0.27)	1700 (0.35)	0.44	0.75	0.36	0.39	0.35	0.14
FS-9	66(0.47)	3100(0.13)	1500 (0.21)	1700(0.18)	0.49	0.64	0.59	0.35	0.33	0.14
FS-11	660(0.4)	2300(0.33)	200(0.32)	54(0.12)	0.55	0.32	0.28	0.27	0.25	0.17
FS-12	770(0.4)	2300(0.33)	220(0.38)	57(0.19)	0.66	0.12	0.33	0.4	0.24	0.46
FS-13	800(0.4)	2500(0.34)	220(0.33)	60(0.14)	0.69	0.54	0.63	0.44	0.32	0.48
FS-14	910(0.42)	2100(0.33)	210(0.33)	67(0.17)	0.81	0.48	0.35	0.34	0.28	0.87
FS-18	610(0.41)	2200(0.33)	180(0.34)	51(0.11)	0.68	0.35	0.65	0.33	0.44	0.43
FS-15	420(0.3)	1200(0.29)	220(0.36)	64(0.14)	0.8	0.48	0.38	0.41	0.47	0.46
FS-16	730(0.39)	2500(0.34)	220(0.36)	54(0.17)	0.69	0.73	0.79	0.38	0.49	0.42
FS-17	620(0.39)	4800(0.34)	670(0.34)	140(0.06)	0.79	0.45	0.57	0.29	0.43	0.46
Mulberrin	250(0.67)	1400(0.28)	170(0.48)	230(0.62)	0.97	0.51	0.94	0.62	0.58	0.96
Nevirapine	97(0.41)	380(0.26)	41(0.3)	210(0.24)	0.73	0.63	0.67	0.65	0.67	0.61
Delavirdine	670(0.5)	1000(0.47)	71(0.32)	580(0.26)	0.8	0.99	0.97	0.41	0.63	0.66

**Table 4:** Chemical structures, IUPAC names and SMILES notation of the eighteen flavonoid compounds used in this study

Compound ID	IUPAC NAMES	SMILES notation	R_3_	R4	R5	R6	R7	R8
FS-1	5-hydroxy-2-(2-hydroxy-3-methoxy-5-sulfophenyl)-4-oxo-4H-chromene-8-sulfonic acid	S(=O)(=O)([O-])C3(=C2(OC(C1(=C(O)C(OC)=CC(=C1)S(=O)(=O)[O-]))=CC(C2=C(O)C=C3)=O))	OCH_3_	H	OH	H	H	SO_3_H
FS-2	3,4-dihydroxy-5-(4-oxo-4H-chromen-2yl) benzenesulfonic acid	S(=O)(=O)([O-])C1(=CC(O)=C(O)C(=C1)C=3(OC2(=C(C=CC=C2)C(C=3)=O)))`	OH	H	H	H	H	H
FS-3	4-hydroxy-3-methoxy-5-(4-oxo-4H-chromen-2-yl)benzenesulfonic acid	S(=O)(=O)([O-])C1(=CC(OC)=C(O)C(=C1)C=3(OC2(=C(C=CC=C2)C(C=3)=O)))	OCH_3_	H	H	H	H	H
FS-4	3-(6-bromo-4-oxo-4H-chromen-2-yl)-4-hydroxy-5-methoxybenzenesulfonic acid	[Br]C3(=CC=2(C(=O)C=C(C1(=C(O)C(OC)=CC(=C1)S(=O)(=O)[O-]))OC=2C=C3))	OCH_3_	H	H	Br	H	H
FS-5	3-(6-chloro-4-oxo-4H-chromen-2-yl)-4-hydroxy-5-methoxybenzenesulfonic acid	ClC3(=CC=2(C(=O)C=C(C1(=C(O)C(OC)=CC(=C1)S(=O)(=O)[O-]))OC=2C=C3))	OCH_3_	H	H	Cl	H	H
FS-6	3-(6-fluoro-4-oxo-4H-chromen-2-yl)-4-hydroxy-5-methoxybenzenesulfonic acid	S(=O)(=O)([O-])C1(=CC(OC)=C(O)C(=C1)C=3(OC2(=C(C=C(F)C=C2)C(C=3)=O)))	OCH_3_	H	H	F	H	H
FS-7	4-hydroxy-3-methoxy-5-(6-methyl-4-oxo-4H-chromen-2-yl)benzenesulfonic acid	S(=O)(=O)([O-])C1(=CC(OC)=C(O)C(=C1)C=3(OC2(=C(C=C(C)C=C2)C(C=3)=O)))	OCH_3_	H	H	CH_3_	H	H
FS-8	4-hydroxy-3-(6-hydroxy-4-oxo-4H-chromen-2-yl)-5-methoxybenzenesulfonic acid	S(=O)(=O)([O-])C1(=CC(OC)=C(O)C(=C1)C=3(OC2(=C(C=C(O)C=C2)C(C=3)=O)))	OCH_3_	H	H	OH	H	H
FS-9	4-hydroxy-3-(7-hydroxy-4-oxo-4H-chromen-2-yl)-5-methoxybenzenesulfonic acid	S(=O)(=O)([O-])C1(=CC(OC)=C(O)C(=C1)C=3(OC2(=C(C=CC(=C2)O)C(C=3)=O)))	OCH_3_	H	H	H	OH	H
FS-10	5-hydroxy-4-oxo-2-(2,3,4-trihydroxy-5-sulfophenyl)-4H-chromene-8-sulfonic acid	S(=O)(=O)([O-])C1(=C(O)C(O)=C(O)C(=C1)C=3(OC2(=C(C(O)=CC=C2S(=O)(=O)[O-])C(C=3)=O)))	OH	OH	OH	H	H	SO_3_H
FS-11	2,3,4-trihydroxy-5-(4-oxo-4H-chromen-2-yl)benzenesulfonic acid	S(=O)(=O)([O-])C1(=C(O)C(O)=C(O)C(=C1)C=3(OC2(=C(C=CC=C2)C(C=3)=O)))	OH	OH	H	H	H	H
FS-12	5-(6-bromo-4-oxo-4H-chromen-2-yl)-2,3,4-trihydroxybenzenesulfonic acid	[Br]C3(=CC=2(C(=O)C=C(C1(=C(O)C(O)=C(O)C(=C1)S(=O)(=O)[O-]))OC=2C=C3))	OH	OH	H	Br	H	H
FS-13	5-(6-chloro-4-oxo-4H-chromen-2-yl)-2,3,4-trihydroxybenzenesulfonic acid	ClC3(=CC=2(C(=O)C=C(C1(=C(O)C(O)=C(O)C(=C1)S(=O)(=O)[O-]))OC=2C=C3))	OH	OH	H	Cl	H	H
FS-14	5-(6-fluoro-4-oxo-4H-chromen-2-yl)-2,3,4-trihydroxybenzenesulfonic acid	S(=O)(=O)([O-])C1(=C(O)C(O)=C(O)C(=C1)C=3(OC2(=C(C=C(F)C=C2)C(C=3)=O)))	OH	OH	H	F	H	H
FS-15	2,3,4-trihydroxy-5-(6-hydroxy-4-oxo-4H-chromen-2-yl)benzenesulfonic acid	S(=O)(=O)([O-])C1(=C(O)C(O)=C(O)C(=C1)C=3(OC2(=C(C=C(O)C=C2)C(C=3)=O)))	OH	OH	H	OH	H	H
FS-16	2,3,4-trihydroxy-5-(6-methoxy-4-oxo-4H-chromen-2-yl)benzenesulfonic acid	S(=O)(=O)([O-])C1(=C(O)C(O)=C(O)C(=C1)C=3(OC2(=C(C=C(OC)C=C2)C(C=3)=O)))	OH	OH	H	OCH_3_	H	H
FS-17	2,3,4-trihydroxy-5-(7-hydroxy-4-oxo-4H-chromen-2-yl)benzenesulfonic acid	S(=O)(=O)([O-])C1(=C(O)C(O)=C(O)C(=C1)C=3(OC2(=C(C=CC(=C2)O)C(C=3)=O)))	OH	OH	H	H	OH	H
FS-18	2,3,4-trihydroxy-5-(6-methyl-4-oxo-4H-chromen-2-yl)benzenesulfonic acid	S(=O)(=O)([O-])C1(=C(O)C(O)=C(O)C(=C1)C=3(OC2(=C(C=C(C)C=C2)C(C=3)=O)))	OH	OH	H	CH_3_	H	H

**Table 5:** Drug-likeness and pharmacokinetic properties calculated for sulphonated flavones with Osiris Property Explorer

Molecule Name	Total Molweight	cLogP	cLogS	HAcceptors	HDonors	Drug-likeness	Mutag-enic	Tumo-rigenic	Reproductive Effective	Irritant
FS-1	442.4	-4.3424	-0.382	11	2	-5.4	none	none	none	none
FS-2	333.3	-0.7955	-1.758	7	2	-4.3	none	none	none	none
FS-3	347.3	-0.5198	-2.072	7	1	-4.2	none	none	none	none
FS-4	426.2	0.2054	-2.906	7	1	-6.5	high	none	none	none
FS-5	381.8	0.0862	-2.808	7	1	-4.2	none	none	none	none
FS-6	365.3	-0.419	-2.386	7	1	-6.1	none	none	none	none
FS-7	361.3	-0.1759	-2.416	7	1	-5.8	none	none	none	none
FS-8	363.3	-0.8655	-1.776	8	2	-4.8	none	none	none	none
FS-9	363.3	-0.8655	-1.776	8	2	-6.7	none	none	none	none
FS-10	444.3	-4.9638	0.228	12	4	-5.9	none	none	none	none
FS-11	349.3	-1.1412	-1.462	8	3	-4.7	none	none	none	none
FS-12	428.2	-0.416	-2.296	8	3	-7	high	none	none	none
FS-13	383.7	-0.5352	-2.198	8	3	-4.7	none	none	none	none
FS-14	367.3	-1.0404	-1.776	8	3	-6.5	none	none	none	none
FS-15	365.3	-1.4869	-1.166	9	4	-5.2	none	none	none	none
FS-16	379.3	-1.2112	-1.48	9	3	-4.8	none	none	none	none
FS-17	365.3	-1.4869	-1.166	9	4	-7.2	none	none	none	none
FS-18	363.3	-0.7973	-1.806	8	3	-6.3	none	none	none	none
CP 94707	372.5	2.7355	-6.649	5	0	3.1	none	none	none	none
efavirenz	315.7	3.6614	-7.173	3	1	-20.7	none	high	none	none
Etravirine	439.3	3.9245	-7.145	7	4	-9.4	none	none	none	none
Mulberrin	426.5	5.8983	-4.857	6	4	-0.5	none	none	none	none
Nevirapine	266.3	2.1423	-3.846	5	2	1	none	none	none	none
Rilpivirinezinc	368.4	4.861	-6.946	6	2	-11.9	none	none	none	none
Vitexilactone	378.5	3.3376	-3.916	5	1	-3	none	none	none	none
Delavirdine	456.6	1.8437	-4.777	9	3	8.2	high	none	none	none
